# A novel toolbox for the *in vitro* assay of hepatitis D virus infection

**DOI:** 10.1038/srep40199

**Published:** 2017-01-12

**Authors:** Jing-Hua Zhao, Ya-Li Zhang, Tian-Ying Zhang, Lun-Zhi Yuan, Tong Cheng, Pei-Jer Chen, Quan Yuan, Ning-Shao Xia

**Affiliations:** 1 State Key Laboratory of Molecular Vaccinology and Molecular Diagnostics, School of Life Science & School of Public Health, Xiamen University, Xiamen 361102, PR China; 2 National Institute of Diagnostics and Vaccine Development in Infectious Diseases, School of Life Science & School of Public Health, Xiamen University, Xiamen 361102, PR China; 3 National Taiwan University College of Medicine, National Taiwan University, Taipei 10051, Taiwan

## Abstract

Hepatitis D virus (HDV) is a defective RNA virus that requires the presence of hepatitis B virus (HBV) for its life cycle. The *in vitro* HDV infection system is widely used as a surrogate model to study cellular infection with both viruses owing to its practical feasibility. However, previous methods for running this system were less efficient for high-throughput screening and large-scale studies. Here, we developed a novel method for the production of infectious HDV by adenoviral vector (AdV)-mediated transduction. We demonstrated that the AdV-based method yields 10-fold higher viral titers than the transient-transfection approach. The HDV-containing supernatant derived from AdV-infected Huh7 cells can be used as the inoculum in infectivity assays without requiring further concentration prior to use. Furthermore, we devloped a chemiluminescent immunoassay (HDV-CLEIA) to quantitatively determine intracellular HDAg with a dynamic range of 5–11,000 pg/mL. HDV-CLEIA can be used as an alternative approach to assess HDV infection. The advantages of our updated methodology were demonstrated through *in vitro* HDV infection of HepaRG cells and by evaluating the neutralization activity using antibodies that target various regions of the HBV/HDV envelope proteins. Together, the methods presented here comprise a novel toolbox of *in vitro* assays for studying HDV infection.

Worldwide, more than 350 million people are persistently infected with hepatitis B virus (HBV), some of whom are co-infected with hepatitis D virus (HDV), a satellite virus of HBV that has the same envelope proteins as HBV^1^. Worldwide, chronic infection with hepatitis B is a major cause of liver cirrhosis and hepatocellular carcinoma and HDV superinfection confers additional risk^2^,^3^. Currently, efficient drugs for eradicating both infections are not available and are urgently required^4^,^5^. The HDV genome encodes two major proteins that are referred to as small-HDAg (S-HDAg) and large-HDAg (L-HDAg). The two proteins share an identical N-terminus of 195 amino acids (aa), and L-HDAg has an additional 19 aa at its C-terminus^3^. S-HDAg is essential for HDV RNA replication, whereas L-HDAg is required for virion assembly^6^,^7^,^8^. Three types of glycoproteins are present in the envelope of HBV/HDV virions: (i) the small surface protein (S-HBsAg); (ii) the middle surface protein (M-HBsAg), which differs from HBsAg by an additional 55 aa at the N-terminus (denoted PreS2); and (iii) the large surface protein (L-HBsAg), which contains a further N-terminal extension (approximately 120 aa, denoted PreS1). The PreS1 domain in L-HBsAg and the major hydrophilic region (MHR) in S-HBsAg are two essential determinants of HBV/HDV infectivity^9^,^10^,^11^,^12^.

Because the viral envelopes of HBV and HDV virions are identical, studies of the cellular entry of both viruses can be conducted using the *in vitro* HDV model^13^. In contrast to HBV infection, HDV infection of susceptible cells, including differentiated HepaRG cells and exogenous NTCP-expressing hepatoma cells (HepG2 or Huh7)^14^,^15^, leads to high levels of viral replication (>300,000 copies per cell), which is easily detected by northern blot hybridization. Therefore the *in vitro* HDV infection assay is therefore widely utilized as a surrogate model to study the function of HBV envelope proteins and to evaluate the activity of entry inhibitors^13^. However, the current system is not robust enough for use in high-throughput screening and large-scale studies because such studies require the efficient production of recombinant HDV (rHDV) at high titers and convenient detection of infection. The current method to produce infectious rHDV based on transient transfection is expensive and inefficient. Northern blot is the most widely used method for detecting HDV RNA, which serves as a marker of infection. However, this assay is time consuming and tedious.

To overcome these issues, we developed a novel method for producing infectious HDV virus using adenoviral vector (AdV) transduction-mediated gene transfer. The performance of this new strategy was systematically investigated herein. We also developed several monoclonal antibodies (mAbs) specific for HDAg. Using these new mAbs, we established a quantitative immunoassay that detects intracellular HDAg protein; this assay may be used as an alternative approach for assessing HDV infection. The advantages of using our updated methodology were illustrated by their use in evaluating the effects of anti-HBs mAbs in neutralizing *in vitro* HDV infection of differentiated HepaRG cells.

## Results

### Developments of anti-HDAg mAbs and HDV-CLEIA

**Figure 1:**
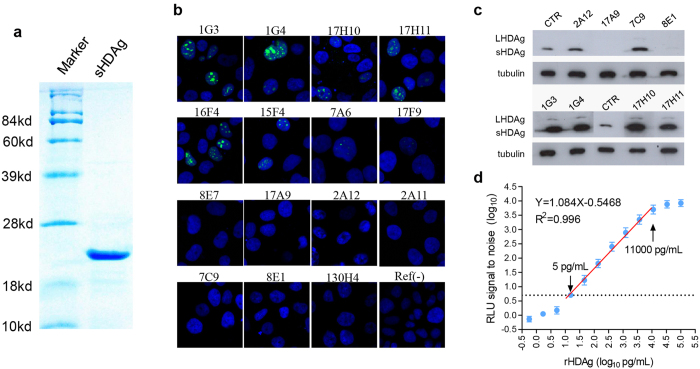
Generation and characterization of mAbs against HDAg for immunofluorescence and chemiluminescence immunoassays. (**a**) SDS-PAGE analysis of purified rHDAg. Immunofluorescence (**b**) and western blot (**c**) detection of the intracellular HDAg of pCD2G-transfected Huh7 cells using the newly developed mAbs. (**d**) Dynamic range of HDV-CLEIA for measuring rHDAg protein. The broken horizontal line indicates the cutoff value that was equivalent to a 5.0-fold RLU signal-to-noise ratio. Four repeats were performed for each concentration. The data were expressed as the mean ± SD. RLU, relative light units; CTR, control Anti-HDAg mAb of D9-3; Ref(−), negative control.

Recombinant S-HDAg was solubly expressed in *E.coli*. The rHDAg, purified by ion-exchange chromatography, was present as a single band on an SDS-PAGE gel with an estimated molecular weight of 24 kDa (Fig. 1a). With rHDAg for immunization, a total of 15 mAbs with binding activity to rHDAg, as determined by ELISA, were developed. Huh7 cells transfected with pCD2G were used to investigate the performance of these mAbs during immunofluorescence detection of intracellular HDAg. As shown in Fig. 1b, nucleoli-concentrated fluorescence signals were present when the 1G3, 1G4, 17H10, 17H11, 16F4, 15F4 and 7A6 mAbs were used. In western blot tests, the 2A12, 7C9, 1G3, 1G4, 17H10 and 17H11 mAbs reacted well with the HDAg of pCD2G-transfected Huh7 cells (Fig. 1c). Compared to the control antibody (a previously developed Anti-HDAg mAb), the 1G3, 1G4, 17H10 and 17H11 mAbs yielded better signals when detecting both S-HDAg and L-HDAg^16^. Using these mAbs, we developed the new HDV-CLEIA method to quantitatively determine HDAg levels. The lower detection limit of HDV-CLEIA was determined to be 5 pg/mL for rHDAg, which was about 10-fold lower than that (50 pg/mL) of current available commercial ELISA assay (Supplementary Figure S1a). The quantitative dynamic range of HDV-CLEIA was 5–11,000 pg/mL (Fig. 1d), which was significantly superior to that of ELISA (50–1,000 pg/mL).

For further evaluation of the assay performance of HDV-CLEIA, differentiated HepaRG cells were challenged with plasmid-transfection derived HDV at 1,000 viral genome equivalents (vge) per cell, the cell samples (1 × 10^5^ cells) were collected at 7 days post-infection (dpi). The intracellular levels of HDV RNA and HDAg in these samples were 1.41 ± 0.08 × 10^8^ copies/mL determined by qRT-PCR and were 12.9 ± 1.7 ng/mL determined by HDV-CLEIA, respectively. The cell lysate samples were 10-fold serially diluted and were detected by HDV-CLEIA, commercial ELISA and qRT-PCR assays. As the results presented in Supplementary Figure S1b, the analytical sensitivity of HDV-CLEIA was about 10-fold higher than that of commercial ELISA, but was about 5-fold lower than that of qRT-PCR.

### Efficient generation of high titers of infectious HDV by AdV transduction

**Figure 2:**
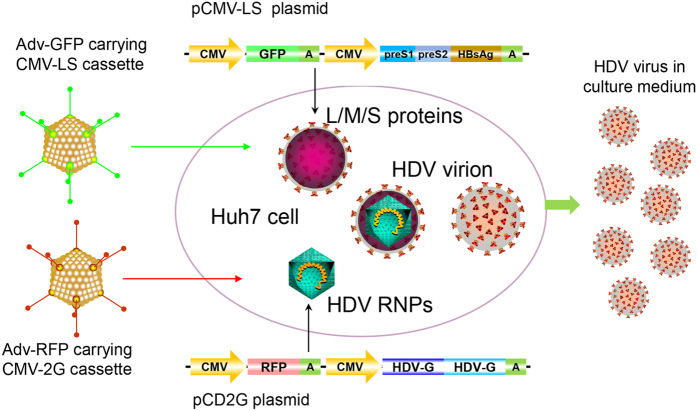
Schematic representation of rHDV production by plasmid transfection and AdV transduction. RNPs, HDV ribonucleoproteins; L/M/S proteins, large, middle and small, respectively, surface proteins of HBV.

**Figure 3:**
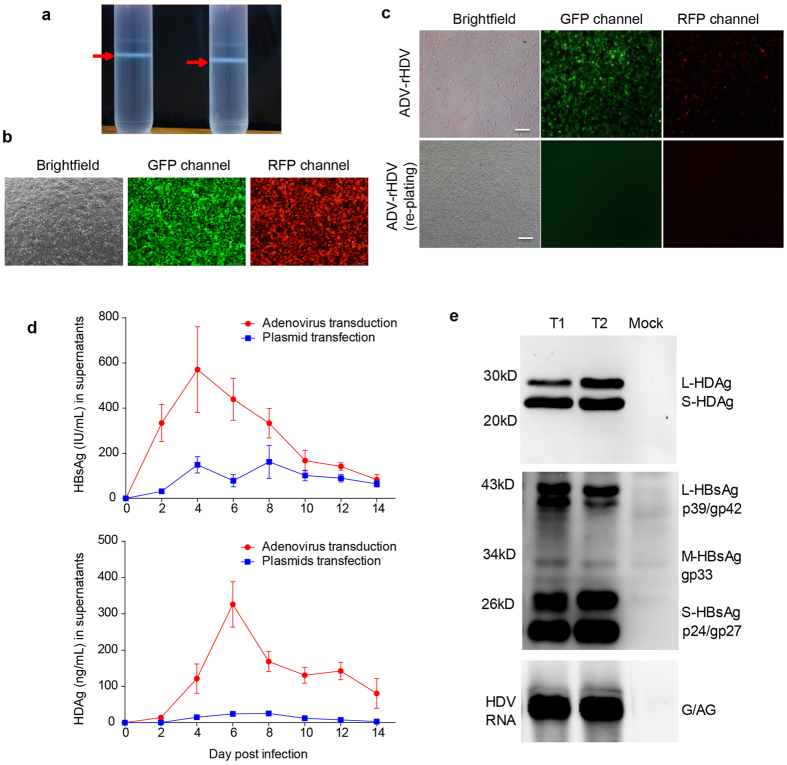
Production and characterization of rHDV by co-transduction with two AdVs. (**a**) Purification of AdVs by CsCl density-gradient ultracentrifugation. The arrowheads indicate the layers containing AdVs. (**b**) Image analyses of Huh7 cells co-transduced with the AdVs of AdGFP-CMV-LS and AdRFP-CMV-HDV2G. AdGFP-CMV-LS carried a GFP-expressing cassette and AdRFP-CMV-HDV2G carried an RFP-expressing cassette. The double positive (GFP+ and RFP+) cells represent cells successfully infected with the two AdVs. (**c**) Image analyses of HepaRG cells infected with AdVs-transduced Huh7 culture supernatants with or without the cell replating procedure. The residual AdVs were indicated by cells that expressed GFP and RFP. (**d**) Profiles of HBsAg (upper panel) and HDAg (lower panel) in the supernatants of Huh7 cells that were transfected or transduced with HDV-packaging DNAs. The data were expressed as the mean ± SD. (**e**) Analyses of viral components in anti-HBs antibody-immunoprecipitated virions from the AdV-derived rHDV stock. Western blots for HDAgs and surface proteins were shown in the top panel and middle panel, respectively. A Northern blot for HDV RNA was shown in the bottom panel. G/AG, genomic/anti-genomic HDV RNA.

Figure 2 illustrates the strategies used to produce HDV by co-transfection and AdV co-transduction in Huh7 cells. According to this approach, we constructed two AdVs carrying HDV-packaging DNA. The two AdVs were produced and purified by CsCl density-gradient ultracentrifugation (Fig. 3a). Successful transduction of the two AdVs could be visualized by fluorescence imaging of RFP and GFP (Fig. 3a). To generate rHDV, the Huh7 cells were co-infected with the two AdVs. One day after AdV transduction, the cells were collected by trypsinization, further washed and replated. This procedure eliminated residual AdV in the supernatant (Fig. 3b), which may interfere with the infection assay. Compared with the transient transfection-based method, the AdV-based approach resulted in significantly higher levels of HDAg (10- to 20-fold higher) and HBV envelope proteins (3- to 4-fold higher) (Fig. 3c). To obtain virus stock for the infection assay, the culture medium of AdV-transduced cells was collected between 6 and 12 days after reseeding. The HDV titer in the cell supernatant was typically approximately 1 to 5 × 10^9^ copies/mL by a HDV RNA qRT-PCR; this titer was approximately 10-fold higher than that resulting from the transient transfection approach. When analyzing viral components in anti-HBs antibody-immunoprecipitated virions derived from the rHDV virus stock, L/M/S-HBsAg and L/S-HDAg and HDV RNA could be simultaneously detected by western blot (L/M/S-HBsAg and L/S-HDAg) and northern blot (HDV RNA) assays (Fig. 3d). Analysis of virus stocks from two independent preparations (Fig. 3d, T1 and T2) showed quite similar results, indicating the reproducibility of this method.

### HDV infection of differentiated HepaRG cells

**Figure 4:**
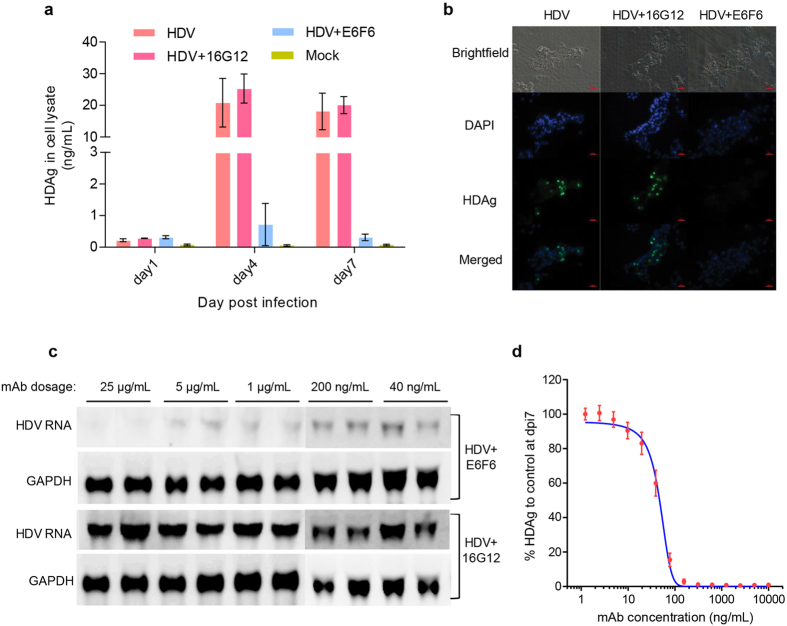
HDV infection of differentiated HepaRG cells using AdV-derived rHDV. Analyses of intracellular HDAg in HDV-infected HepaRG cells by HDV-CLEIA (**a**) and immunofluorescence (**b**, at 7 dpi). (**c**) Northern blot analyses of HDV RNAs of HDV-infected cells in the presence of the neutralizing E6F6 mAb or the 16G12 isotype control mAb at 7 dpi. (**d**) Dose-dependent neutralization curve of the E6F6 mAb for blocking HDV infection in HepaRG cells. The data were expressed as the mean ± SD. The curves were fitted by nonlinear regression (log [inhibitor] vs. normalized response, variable slope).

Differentiated HepaRG cells were used to test the infectivity of AdV-derived rHDV. As shown in Fig. 4a, HDV-CLEIA demonstrated that the intracellular HDAg level was significantly increased at 4 and 7 dpi. The E6F6 mAb is a neutralizing antibody against S-HBsAg, and the 16G12 mAb (specific for HIV P24 protein) was used as an isotype antibody control^17^. In the presence of the E6F6 mAb, the level of HDAg in the lysate of HDV-infected HepaRG cells was greatly reduced, but this level did not change significantly in the presence of the 16G12 mAb (Fig. 4a). Immunofluorescence assay of HDV-infected cells at 7 dpi showed HDAg-positive signals in hepatocyte-like cells (composed of small polarized cells with refractile borders) in the untreated group and the 16G12 mAb-treated group but not in the E6F6 mAb-treated group (Fig. 4b). Moreover, northern blots demonstrated that the E6F6 mAb neutralizing activity effectively reduced the amounts of HDV RNA, even to a concentration as low as 40 ng/mL (Fig. 4c). To quantitatively analyze the neutralizing activity of the E6F6 mAb, HDV challenge with 1,000 vge per cell was performed in the presence of a series of 2-fold dilutions of the E6F6 mAb ranging from 1.22 ng/mL to 10,000 ng/mL, and the HDAg level in cell lysates was determined by HDV-CLEIA at 7 dpi. The dose-dependence curve shown in Fig. 4d indicates that the 50% inhibitory concentration (IC_50_ values) of the E6F6 mAb was 47.08 ng/mL (95% CI: 44.04–50.24 ng/mL). These results demonstrated the infectivity of AdV-derived rHDV and suggest that the intracellular HDAg level determined by HDV-CLEIA may serve as an indicator for HDV infection.

In addition, to investigate potential differences in the infectivity between AdV-derived rHDV and transfection-derived rHDV, HepaRG infection assays were performed using viruses produced by the two approaches at the multiplicity of infection (MOI) of 1,000 and 200 vge per cell in the absence or presence of neutralizing E6F6 mAb. As the results presented in Supplemental Figure S2, no significant difference on the levels of HDV RNA (Supplementary Figure S2a) or HDAg (Supplementary Figure S2b) in cell lysates at 7 dpi was observed between the two groups. These results suggested the AdV-derived rHDV had comparable infectivity with transfection-derived rHDV.

### Neutralizing activity of antibodies against HBV/HDV envelope proteins

We further investigated the neutralizing activity of various antibodies against HBV/HDV envelope proteins using the HDV/HepaRG model. A total of 16 mAbs that target different regions of HBV/HDV envelope proteins were selected^17^,^18^. Among these mAbs, the 4D11 and 7H11 mAbs recognize aa 21–47 of PreS1 in L-HBsAg, which is located at the viral cellular receptor (NTCP) binding site^17^. The 2B2 mAb recognizes aa 33–52 of PreS2, which is located at the translocation motif (TLM) of M-HBsAg and L-HBsAg^19^. The 45E9E mAb is specific for epitopes on the internal hydrophilic loop of HBsAg (aa 57–79)^18^. The E6F6, E7G11, G12F5 and 83H12 mAbs recognize epitopes on the “first loop N-terminus” (aa 119–125) of S-HBsAg, whereas the 42B6, A13A2 and 129G1 mAbs bind epitopes surrounding the “second loop” (aa 137–151) of S-HBsAg^17^,^18^. The A2C1, S1A1, S1F1 and 20A2 mAbs recognize conformation-dependent epitopes surrounding the “a” determinant (aa 124–147) in S-HBsAg^18^.

**Figure 5:**
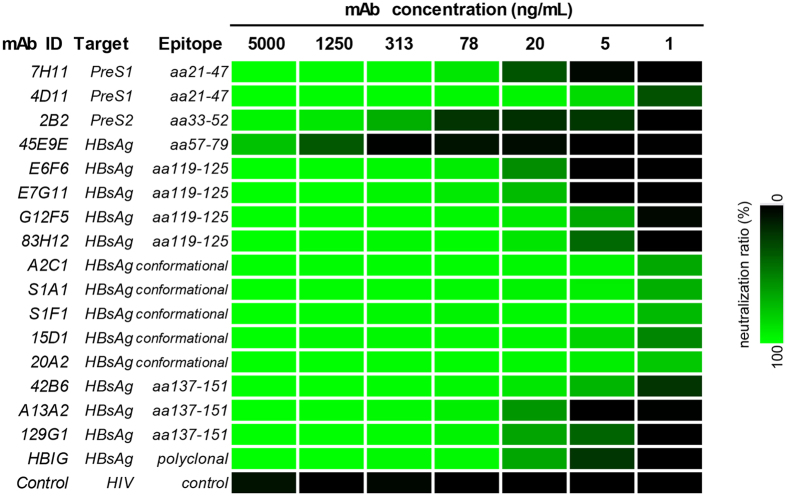
Heat-map illustrating the neutralizing activity of representative mAbs against various regions of HBV/HDV envelope proteins. The map showed the blocking ratios (0 to 100%) of mAbs to intracellular HDAg of HDV-infected HepaRG cell lysates (determined by HDV-CLEIA at 7 dpi), which exhibited a continuous range of colors from black (0) to green (100%). The HBIG was diluted and used according its anti-HBs antibody titer.

HepaRG cells were infected with rHDV in the presence of a series of dilutions of each mAb. The heat-map shown in Fig. 5 illustrates the neutralization profiles of these mAbs at decreasing concentrations. As expected, most of these mAbs were capable of blocking HDV infection. However, the neutralizing activity of 45E9E and 2B2 mAbs was limited and only took effect at comparatively high concentrations (>313 ng/mL for 2B2, and >1250 ng/mL for 45E9E). Among all the mAbs, the 5 conformation-dependent mAbs (A2C1, S1A1, S1F1, 15D1 and 20A2) had the strongest neutralizing activity with an IC_50_ value estimated to be as low as 1–5 ng/mL, which was approximately 10-fold lower than that of HBIG (human polyclonal antibodies against HBV). In the remaining mAbs that bind to linear epitopes, the 4D11 and 42B6 mAbs had a similar neutralizing activity to the conformation-dependent mAbs, whereas the other mAbs (7H11, E6F6, E7G11, G12F5, 83H12, A13A2 and 129G1) had moderate neutralizing activity that was approximately equivalent to that of HBIG.

## Discussion

The *in vitro* HDV infection system is a useful tool for both viral functional studies and drug development for treating HBV/HDV infection^13^. A robust *in vitro* viral infection system should include cells that support viral infection, an efficient method to produce infectious virus and convenient downstream assays to characterize viral infection. Before the identification of a functional receptor for HBV/HDV, HepaRG was the only available continuous cell line supporting HBV/HDV infection^14^. Yan *et al*. identified sodium taurocholate co-transporting polypeptide (NTCP/SLC10A1) as a PreS1 binding-specific receptor in primary tupaia hepatocytes and demonstrated that human NTCP promotes HBV/HDV entry into human hepatoma cells^15^. Subsequently, several exogenous NTCP-expressing hepatoma cells were developed to support *in vitro* HBV/HDV infection^20^. Recently, transgenic mice exogenously expressing human NTCP in the liver were demonstrated to support *in vivo* HDV infection, although the mice were much less susceptible to HBV^21^,^22^. These cells and animals have provided useful models of HDV infection.

Traditional methods to produce HDV for use in the *in vitro* infection assay are mostly based on transient transfection^13^. In this approach, a plasmid expressing HBV surface proteins and a plasmid producing HDV ribonucleoproteins (RNPs) are co-transfected into Huh7 or HepG2 cells to allow co-assembly of surface proteins and HDV RNPs to produce secreted HDV virions. Although the transfection method is widely used, there are several practical disadvantages: (i) the transfection efficiency for Huh7 or HepG2 is limited by available PEI- or liposome-based reagents, resulting in inefficiency output, (ii) commercial transfection reagents are expensive, so cost-effective alternatives must be developed for large-scale preparations, and (iii) additional concentration by PEG precipitation or ultracentrifugation may be required to increase the viral titer before infection. To overcome these issues, baculovirus-mediated transfer of had been used in efficient productions of HDV virus-like particles (VLPs) and antigens in previous studies^23^,^24^. AdV compares favorably to baculovirus system in efficient transduction of mammalian cells, particularly for liver cells. In this study, we developed a novel method for producing infectious HDV based on AdV transduction-mediated gene transfer. As the HDV-producing hepatic cells are highly susceptible to adenovirus infection, AdVs carrying HDV-packaging genes can achieve nearly 100% transduction efficiency in Huh7 or HepG2 cells in the absence of any additional reagents. In our study, the AdV-based method resulted in an approximately 10-fold higher viral titer than that resulting from the transient transfection approach (Fig. 3c,d); therefore, the HDV virion-containing supernatants derived from AdGFP-CMV-LS and AdRFP-CMV-HDV2G co-infected cells can be directly used as the inoculum in infectivity assays without requiring further concentration. Another advantage of our system is the dual color fluorescent reporter (GFP and RFP) system, which allows convenient monitoring of the gene transfer efficiency of the HDV-packaging DNAs and can be used to evaluate any potential residual AdV in the cell culture supernatant. Our data demonstrated that the cell re-seeding procedure at 24 h after incubation with AdVs largely eliminated residual AdVs and minimized any potential interference in downstream HDV infection assays (Fig. 3b). Furthermore, we provided comprehensive data demonstrating that the AdV-derived rHDV was infectious and initiated HDV replication in HepaRG cells (Fig. 4). Note that the construction of vectors used for the production of a given AdV was simplified previously (the AdMax system or Aden-X system)^25^, thus facilitating rapid generation of novel recombinant vectors harboring relevant HDV genomes and/or HBV surface protein variants.

Intracellular HDV RNA detected by northern blot analysis has been widely used as a marker for successful HDV infection. However, this method is time consuming and requires tedious processes for RNA extraction and hybridization; thus, it is not suitable for high-throughput detection in drug screening. In this study, we developed 15 mAbs that specifically recognize HDAg, and several of them (such as 1G3, 1G4, 17H10 and 17H11) were sufficiently able to detect intracellular HDV proteins in western blot and immunofluorescence assays. More importantly, we developed a novel double-sandwich HDV-CLEIA for measuring HDV protein levels. This new assay is highly sensitive and detects HDAgs at concentrations as low as 5 pg/mL. The HDV-CLEIA and northern blot analyses showed comparable results for the neutralizing activity of the E6F6 mAb, which suggests that quantitative measurement of intracellular HDAg by HDV-CLEIA may serve as an alternative approach to assessing HDV infection. A significant advantage of this strategy is that the HDV-CLEIA enables high-throughput detection of HDAg at low levels and minimizes the requirement for sample pretreatment. The improvement in the technology is illustrated by its usefulness in evaluating the neutralizing activity of various antibodies against HBV/HDV envelope proteins (Fig. 5). Among the mAbs tested in this study using the HDV/HepaRG system, 10 of 16 (4D11, 7H11, 2B2, E6F6, E7G11, G12F5, A2C1, 42B6, A13A2 and 129G1) were analyzed in the HBV/HepaRG system in our previous study^17^. As expected, the comparative neutralizing activity of the 10 mAbs in blocking HDV infection significantly correlated with their capability to neutralize HBV infection, as previously reported. Previous studies have demonstrated that the infectivity of HBV/HDV depends on two elements of their envelope proteins: (i) a receptor binding site located within the N-terminus of PreS1 of L-HBsAg, which is responsible for NTCP binding and liver specificity^9^,^15^, and (ii) an infectivity determinant in the surface-exposed “a” antigenic loop in the major hydrophilic region of S-HBsAg, which is responsible for the interaction between virus and cell surface heparin sulfate proteoglycans^11^,^12^. Therefore, most of the mAbs that recognize epitopes in PreS1 and the surrounding “a” determinant of S-HBsAg have moderate to strong neutralizing activities (Fig. 5), whereas the mAbs that bind to other regions (45E9E and 2B2) have only limited blocking capabilities. Notably, among the mAbs we tested, the A2C1 and 20A2 mAbs had the most potent neutralizing activity and are possible candidates for further development of humanized preventative monoclonal antibodies.

In conclusion, the results of our study showed that AdVs carrying HDV-packaging elements could initiate high levels of HDV production following the transduction of Huh7 cells. The AdV-derived rHDV was infectious and could initiate viral replication in HepaRG cells. Quantitative measurement of intracellular HDAg levels by HDV-CLEIA, a method we developed here, may serve as a convenient marker of HDV infection. The methods presented here comprise a robust toolbox that may be used as an *in vitro* HDV infection system, particularly for anti-HDV drug development.

## Materials and Methods

### Plasmids

To generate adenoviral vectors carrying HDV packaging cassettes, PCR was used to amplify the head-to-tail dimeric HDV genomic DNA (HDV2G fragment) from the previously described pCD2G plasmid^26^,^27^ and the HBV surface protein-coding sequence (LS fragment) from the HBV48 plasmid^28^. The HDV2G fragment was cloned into an adenoviral shuttle vector carrying an RFP-expressing cassette (pAd-RFP, Addgene #12520), and the LS fragment was inserted into a GFP-expressing adenoviral shuttle vector (pAdTrackCMV, Addgene #16405). The two fragments are both driven by the CMV promoter. The ligations of DNA fragments into the adenoviral shuttle vectors were performed using the “Gibson Assembly” method^29^. The adenoviral plasmids (AdRFP-CMV-HDV2G and AdGFP-CMV-LS) were generated by homologous recombination between a PmeI-linearized shuttle vector and the supercoiled backbone vector in BJ5183 bacterial cells as previously described^30^.

### Production of virus particles

The AdEasy system was used to generate AdV particles. Briefly, the AdGFP-CMV-LS and AdRFP-CMV-HDV2G plasmids were linearized with the PacI restriction enzyme and then sequentially transfected into HEK293 cells. The primary virus stocks were obtained 7 to 10 days after transfection. To generate higher titer stocks of virus, the HEK293 cells were infected with primary virus stocks at a multiplicity of infection (MOI) of 0.1 to 1. The infected cells were harvested to purify the virus by CsCl density-gradient ultracentrifugation as previously described^30^. The titers of purified virus stocks were determined using a plaque forming assay.

### Proteins and antibodies

To generate recombinant HDV proteins (rHDAg), the encoding DNA of sHDAg from the pCD2G plasmid was ligated into the *E. coli* expression vector pTO-T7^31^. The pTO-T7-sHDAg plasmid was transformed into *E. coli* strain ER2566 for sHDAg expression. Recombinant sHDAg proteins in supernatants obtained from cells that were lysed by sonication were purified in a DEAE-5PW column. Purified sHDAg was used as the immunogen for mAb preparation. Briefly, mAbs were raised in mice using intraperitoneal injection of 100 μg of rHDAg emulsified in Freund’s complete adjuvant, followed by an intravenous booster injection of 20 μg of protein one month later. The resulting hybridomas were screened for the secretion of rHDAg-specific mAbs by ELISA, and positive clones were further expanded and cultured. Cells that produced mAbs were then cloned by at least three rounds of limiting dilution. The culture supernatants of monoclonal hybridomas were collected for protein-A-based purification. Additional mAbs against various regions of HBV/HDV envelope proteins were developed using a similar procedure and were described and characterized in our previous studies^17^,^18^. Hepatitis B immune globulin (HBIG, 100 IU/mL) was purchased from Yuanda Shuyang Pharmaceutical company (Sichuan, China). The total human IgG titer of HBIG was 130 mg/mL determined by human IgG ELISA quantitation kit (Bethyl Laboratories, Inc. Montgomery, TX). The anti-HBs specific antibody titer of HBIG was determined as 283.7 μg/mL by a double-sandwich anti-HBs ELISA quantitation kit and calibrated using human anti-HBs polyclonal antibodies purified by HBsAg-sepharose affinity chromatography (Wantai, Beijing, China).

### Cell culture and *in vitro* HDV infection

HepaRG cells were used for *in vitro* HDV infection. As described previously, the cells were initially cultured in maintenance medium for 2 weeks^13^,^14^. After a 2-week culture, 2% dimethyl sulfoxide (DMSO) was added to the culture medium to induce hepatocyte-like cell differentiation over 2 additional weeks. For the infection assay, differentiated HepaRG cells were incubated with virus stock supplemented with 4% polyethylene glycol 8000 for 24 h at 37 °C. At the end of the incubation, the cells were washed three times with fresh culture medium and further cultivated for analysis. The neutralization assay using the abovementioned antibodies was performed as previously described^17^.

### Northern blot, western blot, RT-PCR and immunofluorescence to assay HDV markers

Northern blot analyses for intracellular HDV RNA were performed using a DIG-labeled probe encompassing the HDV genome as described in a previous study^8^. Immunofluorescence analysis for HDAg was performed according to standard procedures using the 17H11 mAb. To assess virion-associated components, the HDV in the supernatants was initially immunoprecipitated with anti-HBs mAb-labeled magnetic beads. The beads containing immunoprecipitated virions were washed and subsequently subjected to western blot (for envelope proteins and HDAg using the 45E9E and 17H11 mAbs, respectively) and northern blot assays. To determine viral titers, quantitative RT-PCR was performed using HDV-specific primers as previously described^32^.

### Quantitative evaluation of HDAg

To quantitatively determine HDAg levels, a novel chemiluminescent microplate enzyme immunoassay (HDV-CLEIA) was developed in this study. In this assay, the 17H11 mAb was coated on a microplate, and the 1G3 mAb was conjugated to horseradish peroxidase. Cell lysates (20 μL) were initially mixed with dilution buffer (80 μL), the diluents were added to the reaction wells, and the plates were incubated for 60 min at 37 °C, followed by washing and reaction with HRP-conjugated 1G3 mAb. The relative light unit (RLU) values were measured using an Orion II Microplate Luminometer (Berthold, Germany) after the addition of SuperSignal ELISA Pico Chemiluminescent Substrate (Thermo Scientific, Rockford, USA). The sample concentration was calculated by comparing the RLU value with the reference standard of recombinant HDAg. Commercial ELISA kits for HDAg measurements were purchased from Wantai Company (Wantai, Beijing, China) to serve as a control assay.

## Additional Information

**How to cite this article**: Zhao, J.-H. *et al*. A novel toolbox for the *in vitro* assay of hepatitis D virus infection. *Sci. Rep.* **7**, 40199; doi: 10.1038/srep40199 (2017).

**Publisher's note:** Springer Nature remains neutral with regard to jurisdictional claims in published maps and institutional affiliations.

## Supplementary Material

Supplementary Figures
