# Supplementary material for: A novel toolbox for the in vitro assay of hepatitis D virus infection
Source: Sci Rep. 2017 Jan 12;7:40199. doi: 10.1038/srep40199 (PMC5228157; doi:10.1038/srep40199)
Supplement: Supplementary Figures [file srep40199-s1.pdf]

A novel toolbox for the in vitro assay of hepatitis D virus infection

Supplementary information

Jing-Hua Zhao[1,2,*], Ya-Li Zhang[1,2,*], Tian-Ying Zhang[1,2], Lun-Zhi Yuan[1,2], Tong Cheng[1,2], Pei-Jer Chen[3], Quan Yuan[1,2] and Ning-Shao Xia[1,2]
Jing-Hua Zhao[1,2,*], Ya-Li Zhang[1,2,*], Tian-Ying Zhang[1,2], Lun-Zhi Yuan[1,2], Tong Cheng[1,2], Pei-Jer Chen[3], Quan Yuan[1,2] and Ning-Shao Xia[1,2]

[1] State Key Laboratory of Molecular Vaccinology and Molecular Diagnostics, School of Life Science & School of Public Health, Xiamen University, Xiamen 361102, PR China

[2] National Institute of Diagnostics and Vaccine Development in Infectious Diseases, School of Life Science & School of Public Health, Xiamen University, Xiamen 361102, PR China

[3] National Taiwan University College of Medicine, National Taiwan University, Taipei 10051, Taiwan

[*] These authors contributed equally to this work.

Supplementary Figure S1. Evaluation of the analytical sensitivities of different methods in HDV detection.

Supplementary Figure S2. Comparison of the infectivity of transfection-derived and AdV-derived HDV viruses

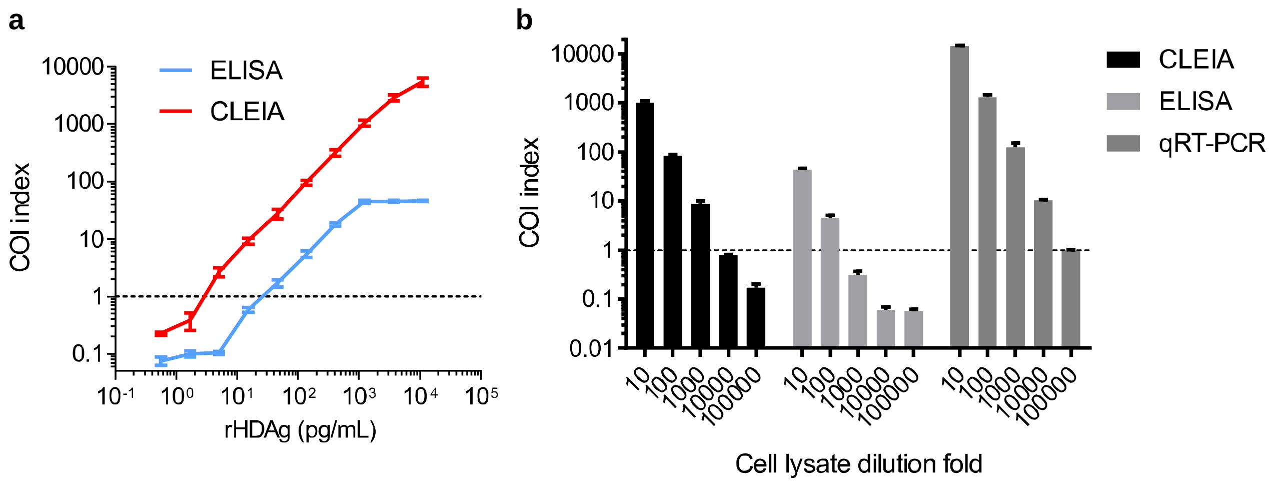

Figure S1. Evaluation of the analytical sensitivities of different methods in HDV detection. (a) Comparison of the performance of the HDV-CLEIA and commercial ELISA for measuring rHDAg protein. (b) Comparison of the performance of the HDV-CLEIA, commercial ELISA and qRT-PCR in detection of a series of 10-fold dilutions of HDV-infected cell lysates (1000 vge, at 7 dpi). COI, cut-off index (the ratio of assay signal to cut-off value), COI values greater than or equal to 1.0 are considered as positive reactions. The HDAg lower detection limit of HDV-CLEIA was 5 pg/mL, and it was about 50 pg/mL for commercial ELISA. The lower limit of detection for qRT-PCR in detection of HDV RNA was about 1.0×10^3 copies/mL.

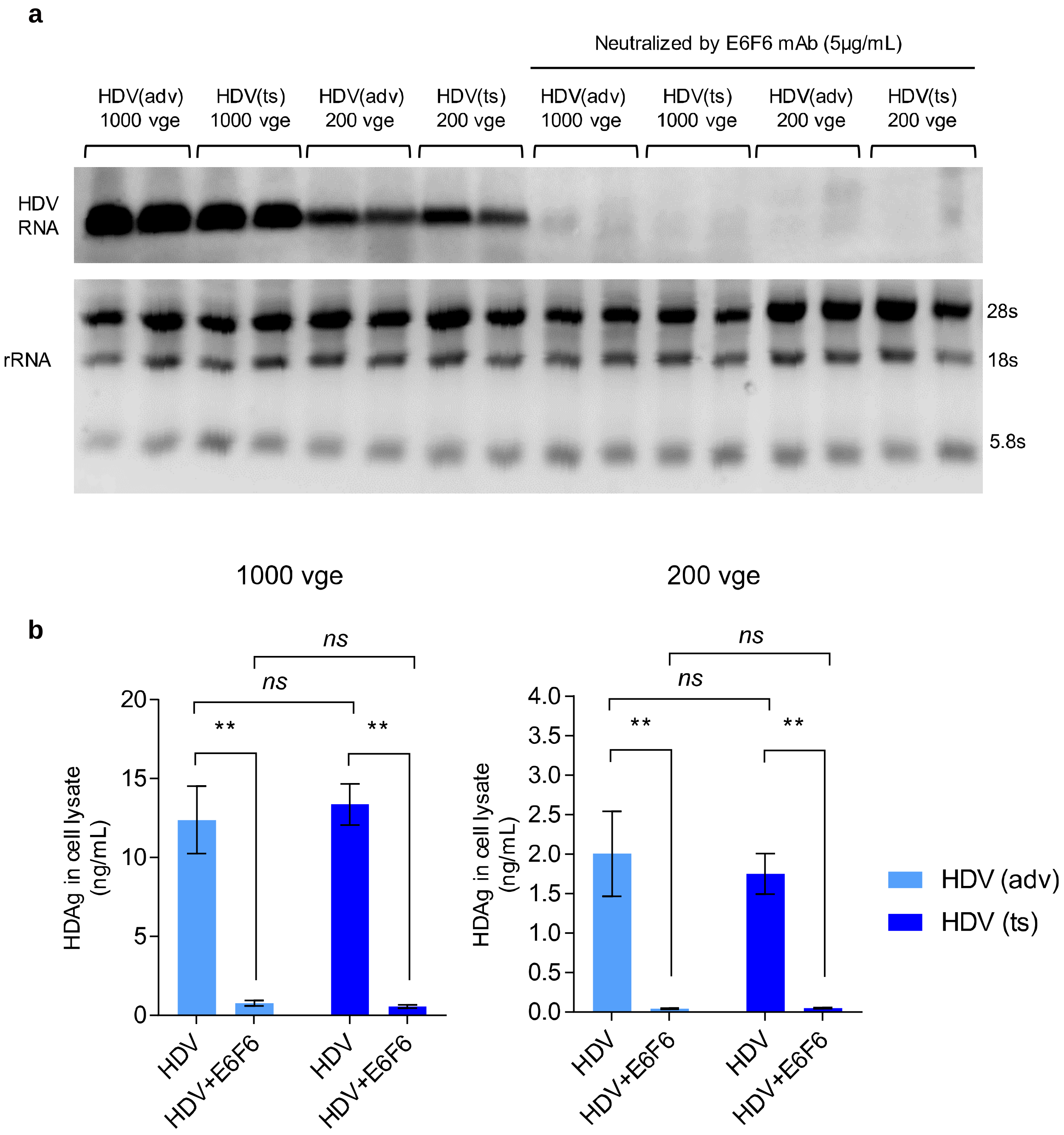

Figure S2. Comparison of the infectivity of transfection-derived and AdV-derived HDV viruses. (a) Northern blot analyses and (b) intracellular HDAg quantifications of HepaRG cells infected by HDV viruses produced by plasmid-transfection and AdV-transduction at 7 dpi. HDV(adv), AdV-derived rHDV; HDV(ts), Transfection-derived rHDV; vge, viral genome equivalents; E6F6 is a viral neutralizing antibody. The HDV infection assays were performed in both presence and absence of E6F6.
